# Effects of the Neuropeptides Pituitary Adenylate Cyclase Activating Polypeptide and Vasoactive Intestinal Peptide in Male Fertility

**DOI:** 10.3390/medicina60040652

**Published:** 2024-04-18

**Authors:** Roba Bdeir, Maha S. Al-Keilani, Khaldoun Khamaiseh

**Affiliations:** 1 Department of Allied Health Sciences, Faculty of Nursing, Al-Balqa Applied University, P.O. Box 206, Al-Salt 19117, Jordan; 2 Department of Clinical Pharmacy, College of Pharmacy, Jordan University of Science and Technology, P.O. Box 3030, Irbid 22110, Jordan; mskeilani@just.edu.jo; 3 Department of Obstetrics & Gynecology, Faculty of Medicine, Al-Balqa Applied University, P.O. Box 206, Al-Salt 19117, Jordan; khamaiseh@bau.edu.jo; 4 Faculty of Medicine, Al-Balqa University, P.O. Box 206, Al-Salt 19117, Jordan

**Keywords:** seminal plasma, sperm parameters, vasoactive intestinal peptide, pituitary adenylate cyclase activating polypeptide, neuropeptides, infertility

## Abstract

*Background and Objectives*: The neuroendocrine system plays a crucial role in regulating various bodily functions, including reproduction, with evidence suggesting its significant involvement in male fertility and sperm development. Vasoactive intestinal peptide (VIP) and pituitary adenylate cyclase activating polypeptide (PACAP) are expressed in both male and female reproductive tissues, influencing penile erection and regulating steroidogenesis in males. Therefore, our study aimed to compare the protein levels of VIP and PACAP in seminal plasma between healthy controls and sub-fertile patients. Additionally, we sought to correlate the levels of these biomarkers with clinical, functional, and laboratory findings in the participants. *Materials and Methods*: The study included a total of 163 male participants for analysis. The participants were further stratified into subgroups of fertile and sub-fertile men of four subgroups according to the 2021 WHO guidelines. Seminal plasma concentrations of the neuropeptides VIP and PACAP were measured using human enzyme-linked immunosorbent assay technique. *Results*: The findings showed statistically significant differences in total sperm count, sperm concentration, total motility, and vitality (*p* < 0.001) between the fertile group and the sub-fertile group. Specifically, significant differences found between healthy males and oligoasthenospermic patients (*p* = 0.002), and between asthenospermic and oligoasthenospermic patients (*p* = 0.039). An ROC analysis showed associated sensitivity and specificity values of 62.2% and 55.6%, respectively, to PACAP seminal levels differentiated between sub-fertile patients from fertile males (*p* = 0.028). No significant difference in seminal levels of VIP was found between the sub-fertile and fertile groups. *Conclusions*: Previous research leads to the point of PACAP active involvement in spermatogenesis. In accordance to our study, in human semen samples, we have seen a significance change in PACAP levels amongst patients with low sperm count or with both low sperm count and low motility, hinting at its contribution and acting as a possible factor in this complex process. Thus, alterations in the levels or actions of these neuropeptides have been associated with certain reproductive disorders in males.

## 1. Introduction

Infertility is a complex medical condition that affects the reproductive system. It is characterized by the inability to conceive after 12 months or more of trials of regular unprotected sexual intercourse to achieve pregnancy [1,2]. Several studies were conducted to understand the underlying etiology where various environmental, biochemical, and genetic factors were found to have association [3,4]. Recently, the World Health Organization (WHO) revealed that approximately 48 million couples worldwide are infertile, where male factor infertility accounts for 20–70 percent of cases depending on the region [5]. While currently there are no explicit published percentages on male infertility in Jordan, it is estimated in the Middle East region for male factor infertility to reach around 60–70% of the cases among couples [6]. Thus, studies on male infertility in Jordan serve to improve the public understanding about the prevalence and seriousness of the issue, guide resource allocation and healthcare planning, and facilitate international collaboration in addressing this important reproductive health issue. Clear statistics on male infertility can guide research efforts. Researchers may focus on understanding the underlying causes, developing new treatment modalities, or improving existing diagnostic techniques based on the prevalence and specific characteristics of male infertility in the population.

The neuroendocrine system is responsible for regulating a number of functions in our body, including reproduction. Specifically, neuropeptides have been found to be involved throughout several phases of reproduction, from sexual behavior, conception, lactation, and parent–infant bonding, as their receptors are found throughout both the male and female reproductive tracts [7,8,9]. While most of the research investigated the role of numerous neuropeptides on female fertility and reproduction, there is evidence indicating their critical involvement in male fertility and sperm development [9,10,11]. Furthermore, some neuropeptides are found in both seminal plasma and follicular fluid indicating their involvement in fertilization [12,13].

Vasoactive intestinal peptide (VIP) and pituitary adenylate cyclase activating polypeptide (PACAP) are expressed in both male and female reproductive tissues, influencing penile erection and regulating steroidogenesis in males [14,15,16]. VIP nerves are distributed throughout the male genital tract and as they responsible for penile erection via stimulating relaxation of vascular and cavernous smooth muscles [17]. VIP nerve dysfunction was associated with sexual dysfunction, and impotent men had reduced VIP levels in their penises [17]. In vivo studies in rats revealed that VIP has protective effects on testicular tissue from detorsion injury through inhibiting the mast cell degranulation and increasing their heparin content [18].

A study by Yan et al., 2020, showed that PACAP enhanced reproduction in obese infertile mice through inhibition of p53-induced apoptosis and the activation of silent information regulator 1 (Sirt1), a deacetylase enzyme responsible for inhibiting the proapoptotic effects of p53 [19]. Moreover, knockout of PACAP in male mice was associated with impaired steroidogenesis [20], and it affected sperm head morphology [21].

Pituitary PACAP plays a role in the gonadotropic hormone secretion [22], where its overexpression in the anterior pituitary of transgenic male mice delayed puberty and negatively affected the levels of gonadotropins and testosterone [23,24].

Overall, it is clear that neuropeptides play integral roles in all aspects of mammalian reproduction and several studies demonstrated their role in sperm function and male fertility [25]. Further investigation is necessary to address the various gaps that remain in our comprehension of these neuropeptides, such as understanding its interaction with its receptor, the hormonal control of their expression, defining its expression in specific cell types within the gonads, especially for VIP, examining the interplay between GnRH and these neuropeptides in regulating gonadotropin expression, and understanding the local effects of PACAP within the reproductive system. But before all that, there is no reference for their local seminal levels, nor do we know if there is a significant difference between healthy fertile vs. sub-fertile samples. Thus, this leads to our research objective to investigate their seminal protein levels, which will set up the foundations for the future research listed above.

These molecules could serve as biomarkers to improve the diagnosis of different infertility-related diseases in men or as new therapeutic targets to treat male infertility. Problems such as defects in spermatogenesis or alterations in gonadal steroidogenesis, as well as alterations found in semen analysis, including asthenozoospermic and oligozoospermic patients, could be addressed in the future with treatments targeting specific neuropeptides. Specific neuropeptides in seminal plasma have the potential to impact diagnostic procedures by serving as biomarkers for fertility issues and influencing treatment strategies through their roles in reproductive physiology and potential as therapeutic targets. Further research in this area could lead to advancements in both diagnostic techniques and treatment options for patients with reproductive health concerns.

Despite the usefulness of conventional semen analysis in the diagnosis of male infertility, about 15% of cases have normal semen parameters indicating the need for further understanding of the pathophysiology of the disease [26]. Seminal plasma is the major source for proteins from the male reproductive glands and is considered a promising noninvasive diagnostic tool for male infertility. To our knowledge, this is the first study to investigate the seminal protein levels of VIP and PACAP. Furthermore, we compared the protein levels between patients with asthenozoospermic and oligozoospermia and healthy fertile men (normospermia). Additionally, we wanted to identify correlations between protein levels and clinical, functional, and laboratory findings of patients.

## 2. Materials and Methods

### 2.1. Study Population and Sample Collection

Between 18 October 2022 and 14 February 2023, seminal specimens were collected from both fertile and sub-fertile men visiting the Assisted Reproductive Technologies unit at Prince Rashid Ben Al-Hasan Military Hospital and Al-Qudah laboratories in Irbid province, Jordan, with age range 23–45 year, by masturbation after a minimum of 72 h of sexual abstinence. Utilizing a power analysis specifically tailored for correlations, and assuming a power of 0.80 along with a significance level of 0.05, with an average population correlation (effect size) of 0.20 as per Polit and Beck (2016) [27], the intended sample size for our study was determined to be 194 participants.

In the present analysis, due to the limited time frame and budget of our project, a total of one hundred and sixty-three male participants were evaluated and divided into two groups: normozoospermic healthy men (control group, n = 81) and aged-matched sub-fertile men (n = 82). The study protocol was approved by the Institutional Review Board of King Abdullah University Hospital-Jordan University of Science and Technology, Irbid, Jordan, with IRB approval reference number: 100/147/2022.

Patient recruitment was carried out in person by a research assistant with all males visiting the center and who have agreed to participate and met the inclusion criteria. No specific recruitment strategies were used. However, having two centers to recruit patients facilitated the attainment of a sample size close to the intended target and ensured the inclusion of a representative sample encompassing both fertile and sub-fertile patients.

The study purpose and procedure was discussed with each patient as well as an informed consent was obtained from each patient. Participants were reviewed with a questionnaire to record patient history, medications, demographic data, marital status, and family history of infertility. Non-eligible patients were excluded from the study. Exclusion criteria included having a family history of infertility, having reproductive problems such as varicocele, testicular tumors, or any chronic illnesses, being on chronic medications, or taking vitamin supplements. For example, smoking has been linked to decreased fertility by impairing sperm quality and reducing sperm count in men. Chronic illnesses such as diabetes and hypertension can damage blood vessels and reduce blood flow to the genitals, leading to erectile dysfunction and decreased fertility, while chronic kidney or liver diseases can lead to hormonal imbalances and reduced testosterone production, which can affect sperm production and fertility. Additionally, kidney dysfunction can impair the body’s ability to eliminate toxins, leading to an accumulation of harmful substances that can affect sperm health. Thus, it was crucial to exclude these cases to ensure that the observed effects are not confounded by external variables.

### 2.2. Semen Analysis

Patients were diagnosed and categorized according to the 2021 WHO guidelines [1]. Using these criteria of semen analysis, the participants were further stratified into fertile group and four sub-fertile groups as follows:

1. Fertile Men:
  - Defined by having a sperm concentration of ≥20 × 10^6^ cells/mL and sperm motility of >40%.

2. Sub-fertile Men:
  - Oligospermia: Men with a low sperm count of <15 million.
  - Asthenospermia: Men with sperm motility of <40%.
  - Oligoasthenospermia: Men with both low sperm count of <15 million and low motility of <40%.

Azoospermia: Men with no sperm in the ejaculation.

During the initial 30 min period after semen collection, samples were examined for viscosity and volume. Subsequently, during the subsequent 30 min, samples were assessed for sperm count, motility, and morphology. Analyses were conducted by a trained andrologists following the WHO protocols/standards [1]. If complete liquefaction does not occur within 60 min, it was recorded; however, in our samples, all were liquefied before then. Normal semen volume was considered for samples with >1.5 mL. Using the hemocytometer, two sets of 200 sperm are counted to determine sperm concentration and sperm count.

For the assessment of sperm motility, approximately 10 µL of each sample were utilized to evaluate both progressive and total sperm motility by using a Makler counting chamber from Irvine Scientific and phase-contrast optics set at 200× g. Spermatozoa exhibiting linear or circular movement were categorized as progressive. Additionally, scanned fields were chosen randomly to minimize assessment bias. Asthenozoospermia was assigned to samples with reduced motility by having less than 40% total sperm motility.

Sperm viability was assessed using the one-step eosin–nigrosin staining test [28]. Sperm showing dark pink or red heads, indicating eosin penetration, were classified as nonviable (dead), whereas those with white heads were considered viable. Low vitality was assigned to samples having more than 42% nonviable.

The assessment of sperm morphology was performed Giemsa stain of dried smear specimen of sperm with 1000× g. Samples must have >4% morphologically normal spermatozoa to exclude teratozoospermia.

The remaining semen samples was centrifuged at 2500× *g* for 10 min. The seminal plasma was kept frozen and stored without preservatives at −80 °C until assayed for neuropeptide levels.

### 2.3. Measurement of VIP and PACAP Levels in Seminal Plasma

Seminal plasma levels of the neuropeptides VIP (CLOUD-CLONE CORP, Houston, TX, USA, cat. # CEA380Hu) and PACAP (CLOUD-CLONE CORP, Houston, TX, USA, cat. # CEB347Hu) were measured using human enzyme-linked immunosorbent assay (ELISA) technique. The absorbance was measured at the appropriate wavelength using EL×800 plate reader (Bio-teak instruments, Winooski, VT, USA). Serum levels of VIP are expressed in pg/mL, and of PACAP in fg/mL. All samples for seminal plasma levels were run in duplicates.

### 2.4. Statistical Analysis

The participants’ data were coded and entered into SPSS (version 19). Continuous variables were presented as mean ± SEM, while categorical variables were presented as numbers and percent values. The differences in patients’ data were analyzed using student’s *t*-test or one-way ANOVA, Chi-square test, or Fisher-Exact test as appropriate. Biomarkers that indicated a significant difference between healthy controls and infertile males were analyzed using receiver operating characteristic (ROC) curves to determine their diagnostic performance in distinguishing between the two groups. After that, the cut-off value was obtained using a method described by Unal [29]. Then, the patients were separated into two groups, and Chi-square test was performed to calculate the positive and negative predictive values, and binary regression analysis to determine the odds ratio and confidence interval. Statistical significance was considered as *p* < 0.05.

## 3. Results

### 3.1. Subjects’ Demographic Characteristics, Semen Criteria, and Protein Levels

A total of 82 sub-fertile patients and 81 age-matched fertile males were recruited for this study (Table 1). The mean age of the patients was 31.90 (±0.55) years, while the mean age of the controls was 31.63 (±0.54) years. About 90% (74/82) of patients were overweight or obese. Semen samples were analyzed according to the 2021 WHO guideline criteria.

**Figure 1:**
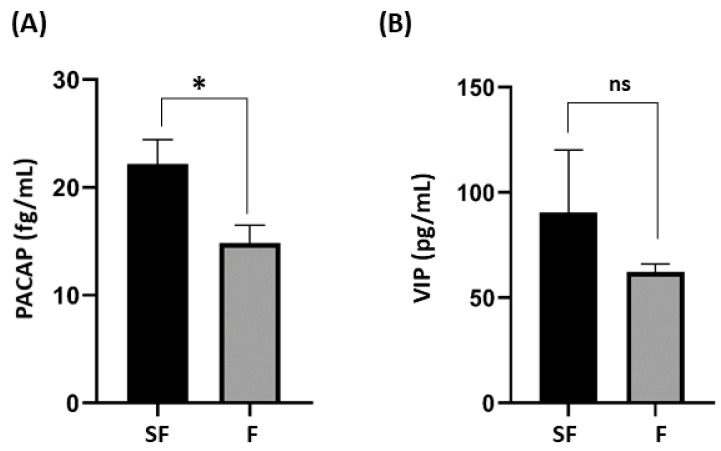
PACAP and VIP seminal levels in sub-fertile group versus fertile healthy males. The seminal levels of two neuropeptides among two groups (SF: sub-fertile male; F: fertile male) are shown. (**A**) PACAP: pituitary adenylate cyclase-activating peptide; * *p* = 0.011. (**B**) VIP: vasoactive intestinal peptide; ns = non-significant. *p*-values were calculated with an independent *t*-test.

As shown in Table 1, the results from the group of fertile males and those of sub-fertile patients showed that there are statistically significant differences in total sperm count, sperm concentration, total motility, and vitality (*p* < 0.001) between the fertile group and the sub-fertile group. Figure 1 reveals the seminal levels of PACAP were significantly higher in sub-fertile patients than in fertile males (*p* = 0.011). No significant difference in seminal levels of VIP was found between the sub-fertile and fertile groups. The average intra-assay coefficient of variation between duplicates for sub-fertile group for PACAP was 5.5%, while that for VIP was 7%; for the fertile group for PACAP, it was 5.3%, while for VIP, it was 4.9%. No inter-assay coefficient of variation since samples were run in duplicates in the same plate, thus no plate-to-plate variation.

**Figure 2:**
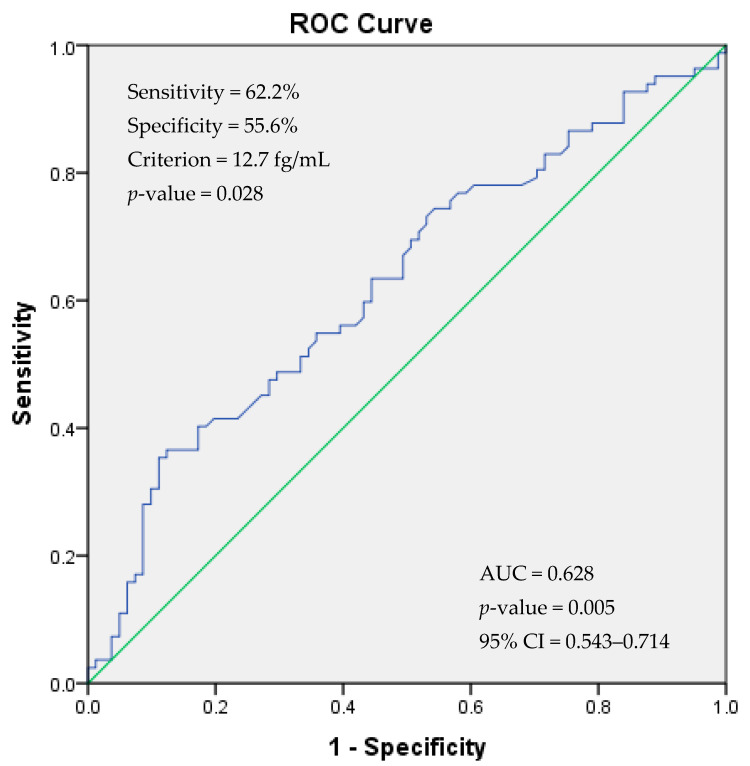
Receiver operating characteristic (ROC) curve showing cut-off of PACAP seminal level for the diagnosis of male infertility. PACAP: pituitary adenylate cyclase-activating peptide; AUC: area under the curve.

According to a method proposed by Unal [29], we determined a cut-off value of 12.7 fg/mL for the PACAP seminal level to differentiate between the fertile and sub-fertile groups, with associated sensitivity and specificity values of 62.2% and 55.6%, respectively. The positive and negative predictive values were calculated as 58.6% and 59.2%, respectively. PACAP seminal levels more than or equal to 12.7 fg/mL differentiated sub-fertile patients from fertile males (*p* = 0.028) with an odds ratio of 0.628 (95% CI = 0.543–0.714, *p* = 0.005; Figure 2).

### 3.2. Comparison between Sub-Fertile Patients’ Groups and Fertile Males’ Group

As shown in Table 2, sub-fertile patients were divided according to sperm count and motility into four groups: oligospermic (low sperm count, normal motility), asthenospermic (normal sperm count, low sperm motility), oligoasthenospermic (low sperm count, low sperm motility), and azoospermic (no sperms). An analysis revealed significant differences in semen analysis between the groups in terms of total sperm count, semen volume, sperm concentration, total motility, presence of normal forms, and vitality.

**Figure 3:**
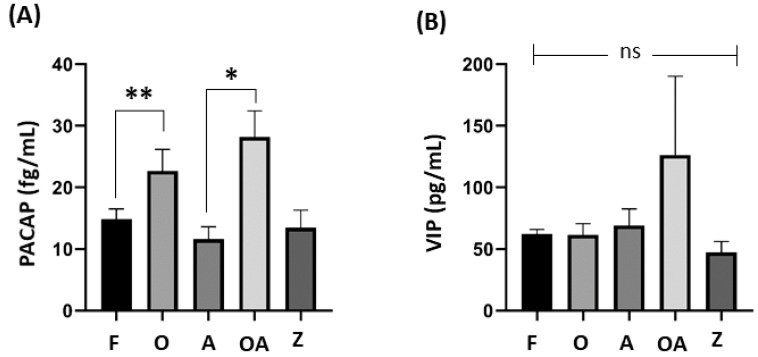
**Comparison of PACAP and VIP seminal levels between sub-fertile groups and fertile healthy males.** One-way ANOVA test followed by Tukey test to find significant differences between groups among (**A**) PACAP seminal levels, where significant is noted by * *p* = 0.002 and ** *p* = 0.039, while no bar was not significant, and (**B**) VIP seminal levels, where ns (= not significant) differences were found amongst all groups. *p* ≤ 0.05 was considered statistically significant. PACAP: pituitary adenylate cyclase-activating peptide; VIP: vasoactive intestinal peptide; F: fertile healthy males. Sub-fertile males were further stratified into four groups: O: oligospermic patients; A: asthenospermic patients; OA: oligoasthenospermic patients; Z: azoospermic patients.

As shown in Figure 3, significant differences in seminal level of PACAP was also found between healthy males and oligoasthenospermic patients (*p* = 0.002), and between asthenospermic and oligoasthenospermic patients (*p* = 0.039).

### 3.3. Associations between Seminal Levels of VIP and PACAP and Seminal Parameters of Sub-Fertile Patients

As shown in Table 3, in sub-fertile patients, statistically significant positive correlations were found between PACAP seminal level and liquefaction time (*p* = 0.011), normal morphology (*p* < 0.001), and VIP seminal level (*p* < 0.001). No significant correlations were found between VIP seminal level and seminal parameters of patients.

## 4. Discussion

The present study demonstrates the significance of PACAP seminal levels and its indication between healthy and sub-fertile men. All the results point to a significant change in PACAP seminal levels amongst sub-fertile men, in particular oligoasthenospermic and asthenospermic patients as it is positively correlation with liquefaction time and morphology. PACAP is known to be present in the male reproductive system, and it has been implicated in the regulation of spermatogenesis and the sperm functions [30,31,32].

The testes harbor the highest levels of PACAP outside of the nervous system, marking a significant finding that initially linked it to the gonads [33]. Studies have suggested that PACAP play a role in supporting the survival and development of germ cells affecting spermatozoa’s development and functionality. In mouse, it has been demonstrated that PACAP can traverse the vascular segment of the blood–testis barrier, allowing it to actively engage in the reproductive function of the testes and add to the pool of PACAP within the testicular environment [34].

In particular, it has been demonstrated that PACAP primarily resides at the periphery of seminiferous tubules within early germ cells, indicating its likely autocrine regulation during spermatogenesis [35,36]. Furthermore, PACAP influences Sertoli cell activity by stimulating the production of cAMP, as well as the secretion of estradiol, inhibin, and lactate [37]. This effect is attributed to the presence of two cAMP-response-like elements in the human PACAP promoter [38,39]. As the epididymis-derived PACAP is also thought to influence the stages of spermiogenesis [40,41], a more recent study observed a gradual increase in PACAP mRNA levels in the testis and epididymis during puberty in rats, particularly in spermatocytes and round spermatids [42]. Similarly, PACAP and its receptors were found to be coexpressed in the cytoplasm of spermatids [43].

In human testis, PACAP presence was also recorded in spermatogonia and round spermatids [44]; in particular, by analyzing RT-PCR, the authors detected mRNAs for PACAP and its receptors in human testes. While observing immunohistological samples from human testicular tissue and testicular germ cell tumors, seminoma tumor cells showed disseminated PACAP immunopositivity, and embryonal carcinoma cells showed relatively weak immunoreactivity. In contrast, spermatogonia and spermatids from normal testes showed PACAP positivity, suggesting a role in human spermatogenesis [44].

PACAP is also present in the developing acrosome of spermatids, but not in mature sperms. The anterior acrosome of epididymal sperm exhibited mild immunostaining in a research by Tanii and colleagues, indicating that PACAP works on the oocyte at the site of fertilization, promoting sperm penetration [45]. By evaluating the PACAP gene expression using RT-PCR, another study has demonstrated a stage-specific expression of PACAP during the spermatogenic cycle. This analysis revealed high expression in the postmeiotic round spermatids during developmental stages I–VIII [46]. There is a noticeable temporal and stage-dependent expression of PACAP mRNA. This RT-PCR investigation of FSH-R mRNA levels agrees with a previous work that used Northern blot analysis of pooled tubule segment RNA, having the same PACAP mRNA expression pattern and levels [47]. The messenger cAMP produced in response to the effects of FSH and PACAP on Sertoli cells significantly down-regulates the levels of SH-R mRNA [48]. These findings imply that PACAP might be a significant factor in the temporal oscillatin in cAMP levels that are stage-dependent in the rat seminiferous tubule. One discrepancy between studies by Rannikko et al. and Daniel and Habener is that the first-mentioned study observed that the minimal levels of FSH-R mRNA are expressed in stage VI of the rat spermatogenic cycle. One possible explanation could be the challenges associated in precisely staging the tubule segments. In addition, others showed the expression of PACAP and its receptors in germ cells of mouse testis, particularly in spermatocytes I, spermatids, and spermatozoa [49].

Essentially, it has been shown that PACAP may have an endocrine, autocrine, or paracrine role in spermatogenesis. Accordingly, the differences in observed expression between studies may be caused by yet to be identified influencing variables [23]. PACAP may have differing effects depending on its concentration and timing of exposure during spermatogenesis. The optimal levels of PACAP might support normal sperm development and function, while either excessive or deficient levels could lead to disruptions in spermatogenesis and infertility. PACAP could interact with other signaling molecules and pathways involved in spermatogenesis and male fertility. Genetic variations and environmental factors could modulate the effects of PACAP. Under certain pathological conditions or diseases, such as hormonal imbalances, inflammation, or oxidative stress, PACAP signaling pathways might be dysregulated, such as via tumors or sperm parameter defects. Finally, studies across different animal models and human subjects may reveal species-specific effects of PACAP on male fertility. Nonetheless, all these data lead to the point of PACAP active involvement in spermatogenesis. In accordance with our study, in human semen samples, we have seen a significance change in PACAP levels amongst patients with a low sperm count or with both a low sperm count and low motility, hinting at its contribution and acting as a possible factor in this complex process.

PACAP also acts at the hormonal level influencing the synthesis of testosterone by Leydig cells indicating its involvement in steroidogenesis [24]. More specifically, through engaging with type I PACAP receptors in fetal rats, the results suggested that PACAP is a highly potent regulator of fetal testicular steroidogenesis [50]. PACAP involvement in the differentiation of immature mouse Leydig cells has been shown by exerting early stimulatory effect on cAMP formation-steroidogenesis and controlling the course of the cell cycle, inducing a prolonged suppressive effect on cell proliferation in TM3 cells [51]. Through studying PACAP-deficient mice, down-regulating testosterone production prevents the testicles from aging as a result of apoptosis and lowers the generation of reactive oxygen species [20]. A more recent study further looked at PACAP knockout in mice where, in accordance, it showed a disturbed signaling in spermatogenesis, which could be a factor responsible for a reduction in fertility [25].

In parallel with our results of impacted PACAP levels amongst oligoasthenospermic patients, influencing motility is also among the possible functions of PACAP. Gozes and colleagues’ findings, which demonstrated that the addition of a PACAP antagonist peptide resulted in a reduction in motility, offered evidence for PACAP’s potential endogenous stimulatory action to sperm motility [52]. There are reports showing that PACAP could increase the motility of abnormally slow-moving human sperms, while normal moving sperms were not influenced [21,31]. Furthermore, in agreement with our findings amongst both oligoasthenospermic and asthenospermic patients, PACAP- deficient mice were shown to have negative effects of sperm morphology, in particular, having smaller sperm heads than those from wild types [21]. Meanwhile, looking at the PACAP knockout mice, there was a 10% reduction in the normal sperm percentage, with a predominant increase in sperms with detached heads [31]. However, motility can be compensated for by other factors mentioned in our present study, as shown in mice with normal motility and deficiency in PACAP [31], although exogenous PACAP did not alter the sperm motility. In fact, it has been observed that PACAP promotes fertilization in mice through increasing motility and the penetration of ovum [45].

Both PACAP and VIP are considered as neuromodulators with diverse physiological roles, and their presence in the male reproductive system suggests their involvement in maintaining male reproductive health. However, in our current study, we found no significant association between VIP seminal levels amongst sub-fertile human semen samples. Yet, VIP has been shown to be involved in testosterone production and reproductive aging in mouse testis [53,54]. By looking at the whole genome of cattle, bull fertility has been linked to the VIP gene by using a genome-wide association study [55]. As male mice lacking VIP (VIP knockout) exhibited reduced serum levels of FSH and testosterone compared to wild-type mice, the process of testicular aging was delayed, in contrast to that observed in young mice. This suggests VIP may influence testicular aging. Furthermore, the preservation of testicular structure was not as pronounced as observed in PACAP-null mice, and these animals were still fertile [53]. Importantly, research suggests VIP and PACAP exhibit distinct reproductive phenotypes, indicating that these two peptides do not compensate for each other but instead work together to regulate testicular function. Because they share common G-protein-coupled receptors, they elicit many similar effects. Nevertheless, their functional redundancy is not absolute, as a third receptor, referred to as PAC1, exists, which has a selective preference for PACAP over VIP and may serve a crucial role in hindering VIP from entirely compensating for the absence of PACAP, or vice versa. There is enough evidence in the literature linking VIP to fertility; however, it is possible that a larger human sample size, more comprehensive clinical cases by including hormonal imbalance, or a more severe phenotype of sub-fertility problems may further reveal the association between VIP seminal levels between completely fertile cases.

In the current study, we examined the potential of PACAP as a diagnostic marker using ROC analysis. According to a study by Xia et al., an area under the curve (AUC) value equal to one is a perfect value to classify 100% of subjects correctly, whereas an AUC of 0.5 indicates that subjects were randomly classified [56]. As suggested by Xia et al. [56], an AUC value between 0.9 and 1.0 indicates an excellent biomarker; between 0.8 and 0.9, good; 0.7 to 0.8, fair; 0.6 to 0.7, poor; and 0.5 to 0.6, fail. Accordingly, PACAP semen concentration was a poor diagnostic marker of male infertility. Thus, confirmatory prospective studies on a larger infertile male population should be performed to evaluate the accuracy of considering PACAP as a diagnostic biomarker for male infertility.

Understanding the mechanisms by which PACAP and VIP influence male fertility can help in identifying potential biomarkers for diagnosing infertility. For example, alterations in PACAP or VIP levels in biological samples such as blood or semen could serve as indicators of underlying reproductive issues. Additionally, insights into the roles of PACAP and VIP in regulating testosterone production and testicular function could inform the development of novel therapeutic approaches for treating male infertility. Moreover, elucidating the specific pathways through which PACAP and VIP affect spermatogenesis and sperm quality could lead to the discovery of new targets for pharmacological intervention. This could include the development of drugs that modulate PACAP or VIP signaling pathways to improve sperm production or enhance sperm motility and viability. Furthermore, a deeper understanding of how PACAP and VIP interact with other regulatory factors in the male reproductive system could pave the way for personalized treatment strategies tailored to individual patients. For instance, targeting PACAP or VIP signaling pathways in combination with other therapies could offer synergistic effects and improve treatment outcomes for men with infertility issues.

## 5. Conclusions

Our findings consistently indicate a notable alteration in PACAP levels in the seminal fluid of sub-fertile men. PACAP, known to be present in the male reproductive system and implicated in regulating spermatogenesis and sperm functions, was also observed in human testicular cells like spermatogonia and round spermatids. Our study corroborates this by demonstrating significant changes in PACAP levels in semen samples from individuals with low sperm count or both low sperm count and motility, suggesting its involvement in this intricate process.

Prior research has shown that inhibiting PACAP with an antagonist peptide reduces sperm motility, indicating PACAP’s potential endogenous stimulatory effect on motility. Additionally, studies on PACAP knockout models have revealed disrupted signaling in spermatogenesis, implicating PACAP in fertility reduction. Therefore, our results align with previous findings on PACAP’s impact on sperm motility among oligoasthenospermic patients, suggesting a potential role for PACAP in influencing motility.

Both PACAP and VIP are recognized as neuromodulators with diverse physiological functions, including roles in the male reproductive system. However, our study did not find a significant association between VIP levels in seminal fluid and sub-fertility in human samples.

Exploring the practical implications of PACAP and VIP in diagnosing and treating male infertility can not only advance our understanding of their mechanisms of action but also drive the development of innovative strategies for addressing reproductive health challenges in men.

## Figures and Tables

**Table 1:** Descriptive and semen parameters between groups of fertile males and sub-fertile patients.

	Fertile Males (*n* = 81)	Sub-Fertile Patients (*n* = 82)	*p*-Value
Age	31.63 ± 0.54	31.90 ± 0.55	0.724
Height	175.95 ± 0.60	177.49 ± 0.54	0.058
Weight	90.57 ± 1.25	89.87 ± 1.26	0.693
BMI	29.17 ± 0.29	28.46 ± 0.31	0.094
Liquefaction time (min)	29.14 ± 1.11	32.19 ± 1.09	0.052
Total sperm count (10^6^/mL)	56.56 ± 2.83	11.20 ± 2.0	<0.001
Semen volume (mL)	3.83 ± 0.17	3.15 ± 0.38	0.108
Sperm concentration (10^6^/mL)	17.38 ± 1.22	4.46 ± 1.14	<0.001
Total motility (%)	66.96 ± 1.07	23.98 ± 2.81	<0.001
Normal morphology (%)	32.01 ± 3.13	31.00 ± 3.33	0.825
Viscosity	0.28 ± 0.08	0.44 ± 0.09	0.191
Vitality, n (%)			<0.001 *
Normal vitality	79 (97.5)	15 (18.3)	
Low vitality	2 (2.5)	56 (68.3)	
Azoospermia	0 (0.0)	11 (13.4)	
PACAP	14.87 ± 1.6	22.15 ± 2.30	0.011
VIP	62.18 ± 3.76	90.47 ± 29.74	0.350

All data are presented as mean ± SEM (standard error of the mean). * Chi-square test. *n* = sample size PACAP: pituitary adenylate cyclase-activating peptide; VIP: vasoactive intestinal peptide.

**Table 2:** Comparison between the four stratified sub-fertile patients’ groups and fertile males’ group.

	F (n = 81)	O (n = 19)	A (n = 12)	OA (n = 38)	Z (n = 13)	*p*-Value
Age	31.63 ± 0.54	30.42 ± 0.91	31.92 ± 1.25	31.63 ± 0.87	34.85 ± 1.40	0.154
Liquefaction time (min)	29.14 ± 1.11	31.32 ± 2.41	29.58 ± 2.34	34.34 ± 1.52	29.09 ± 3.36	0.108
Total sperm count (10^6^/mL)	56.56 ± 2.83	10.98 ± 4.21	37.75 ± 7.16	6.16 ± 0.87	0	<0.001
Semen volume (mL)	3.83 ± 0.17	4.85 ± 1.44	3.59 ± 0.58	2.62 ± 0.22	1.84 ± 0.60	0.004
Sperm concentration (10^6^/mL)	17.38 ± 1.22	6.26 ± 4.38	12.20 ± 2.41	2.64 ± 0.33	0	<0.001
Total motility (%)	66.96 ± 1.07	56.8 ± 2.41	25.14 ± 3.88	16.59 ± 2.62	0	<0.001
Normal morphology (%)	32.01 ± 3.13	39.11 ± 7.26	21.50 ± 7.37	38.92 ± 4.57	0	0.001
Viscosity	0.28 ± 0.08	0.26 ± 0.13	0.83 ± 0.27	0.39 ± 0.13	0.44 ± 0.24	0.168
Vitality, n (%)						<0.001
Normal	79 (97.5)	11 (57.9)	0 (0.0)	2 (5.3)	2 (15.4)	
Low	2 (2.5)	8 (42.1)	12 (100)	36 (94.7)	11 (84.6)	
PACAP	14.87 ± 1.6	22.68 ± 3.50	11.62 ± 2.01	28.18 ± 4.23	13.48 ± 2.84	0.001 *
VIP	62.18 ± 3.76	61.54 ± 9.03	69.07 ± 13.52	126.46 ± 63.77	47.34 ± 8.78	0.491

All data are presented as mean ± SEM (standard error of the mean) unless otherwise indicated. * Chi-square test. n = number; PACAP: pituitary adenylate cyclase-activating peptide; VIP: vasoactive intestinal peptide. F: fertile healthy males. Sub-fertile males were further stratified into four groups: O: oligospermic patients; A: asthenospermic patients; OA: oligoasthenospermic patients; Z: azoospermic patients.

**Table 3:** Correlation analysis between seminal levels of PACAP and VIP levels with semen parameters in sub-fertile patients.

	Sub-Fertile Patients	
	PACAP	VIP
Age		
r	−0.142	−0.082
*p*-value	0.204	0.465
Liquefaction time (min)		
r	0.282	0.118
*p*-value	0.011	0.295
Total sperm count (10^6^/mL)		
r	−0.189	−0.067
*p*-value	0.094	0.554
Semen volume (mL)		
r	−0.066	−0.047
*p*-value	0.558	0.673
Sperm concentration (10^6^/mL)		
r	−0.135	−0.041
*p*-value	0.226	0.715
Total motility (%)		
r	−0.063	−0.122
*p*-value	0.618	0.331
Normal morphology (%)		
r	0.392	0.130
*p*-value	<0.001	0.251
Viscosity		
r	−0.168	−0.065
*p*-value	0.142	0.570
PACAP		
PCC	--	0.626
*p*-value	--	<0.001
VIP		
r	0.626	--
*p*-value	<0.001	--

PACAP: pituitary adenylate cyclase-activating peptide; VIP: vasoactive intestinal peptide. Linear correlation is measured by r, Pearson correlation coefficient. *p*-values are calculated using two-tailed significance.

## Data Availability

The data underlying this article are available in the article.
